# On an Equal Distribution of Excitement, and of the Circulating Fluids

**Published:** 1822-12

**Authors:** 


ART. VII.—*On an equal Distribution of Excitement, and of the Circulating Fluids.* By Dr. KINGLAKE.

[Concluded from page 397.]

THE disorders induced by irregular excitement and vascular transmission of the circulating fluids, may be of every conceivable variety, according to the degree of the irregularity, and to the nature of the part in which it may occur. Diseased action of the most simple kind, that which consists of a merely increased exertion of vital power, may by delay and progressive changes attain to a morbid state of the most complicated description. The vital power may be insufficient for the tone of health, and yet be so stimulated by morbid disorganization as to keep up an immoderate degree of excitement. Affections proceeding to this length on vital organs are soon destructive of life; but they may exist in textures that are not immediately connected with the vital functions for an indefinite period, and prove a source of unhealing and incessant irritation. The visceral parts, such as the brain, lungs, liver, stomach, intestines, kidneys, &c. have respective offices to execute that require a given degree of excitement, and a certain proportion of circulating fluids, to perform them correctly; but either excess or deficiency falling short of this salutary standard, would furnish a condition of disease that would assume an intensity and character corresponding to the part affected, and to the derangement of function that may be disturbed.

If the brain be affected either by immoderate excitement or by redundant fluids, the functions on which the nervous and mental powers depend will be disordered, and at once involve in difficulty and danger the vital energies of the body and the rational faculties of the mind. The lungs are required to admit, by a secerning or elective process, the oxygenous principle of the atmospheric air, and to detach and exclude a carboniced gas, both of which offices are essential to life. These operations can only be carried on in a healthy manner by extending a due proportion of excitement and of the circulating fluids to the pulmonary structure. Deviations from this state are conditions of disease, liable to such adventitious diversities as circumstances of violence, duration, altered structure, distempered excitability, and constitutional peculiarities, may occasion. The stomach, in its secreting and digesting offices,—the intestines, in their assimilating, chylifactive, and transmitting uses,—the liver, in its biliary and circulating functions,—the kidneys, in removing from the system superfluous water, containing acrid, mucilaginous, saline, and other excrementitious substances, in common with every portion of muscular, vascular, cellular, ligamentous, tendinous, and osseous texture, relatively and equally owe both the conditions of health and disease to the quantity of excitement and of circulating fluid by which they are respectively actuated.

If any part be unduly loaded and obstructed by blood, whether it be arterial or venous, it is obvious that the circulating current must in that part be impeded, and necessarily turned off in collateral channels, that must induce excessive vascular distention and excitement in the situation where the first push is made. Both the obstructed part and the adjoining passages, oppressed by redundant fluids, may be regarded, during the continuance of such obstruction, as being out of the bounds of the circulation; and, therefore, the diminished area through which the circulating fluids have to pass must induce a plethoric fulness throughout the whole sanguiferous system. Surcharged as the larger vessels must be with blood, and gorged as those of a smaller description are with the circulating fluids, when congestive fulness of the viscera occurs, it is not difficult to conceive, in such circumstances of diseased excitement and vascular plenitude, that the various secreting actions, as well as the several vital processes on which the peculiar excitability of the different textures of the animal frame depends, must be disturbed, and may, under such influence, originate diseases to which they are respectively liable, whether nervous, vascular, glandular, muscular, membranous, ligamentous, tendinous, or osseous. There is hardly any imaginable form of disease that may not proceed from an undue distribution of excitement and of the circulating fluids. Like the fluids which circulate through the system, the excitability should be diffused over the frame in such proportions to the different parts as the peculiar offices of such parts may especially require. It is obvious that neither health nor life can be sustained, for even the shortest time, without the motive agency of the circulating fluids. They contain materials necessary to the maintenance of life, and afford, by mechanical bulk, a distending stimulus that essentially contributes to the uniformity and equality of the circulating function. The circulating fluids are supplied by the alimentary canal, and are variously elaborated by the different structures through which they pass, so as to fit them for the relative purposes for which they are designed. The excitability is a property emanating from the condition of life, and is probably derived from vital processes obtaining in every portion of the system, but owing its efficiency to the continuous and co-operative agency with which the same property pervades and actuates the whole frame. It is this diffusive continuity that imparts to the excitable property the capacity of becoming more or less equally distributed,—of being unduly accumulated in some situations, and of being deficient in others, by which its agency or transmitting power is not dissimilar to that which is possessed by the circulating fluids.

The different parts of the animal frame are more or less excitable according as the existing organization may be adapted to generate or evolve a larger or smaller quantity of the capacity for being excited. The brain, heart, and stomach, are pre-eminently endued with excitability, and on common occasions first denote the unhealthy state. In inflammatory or febrile affections, those organs necessarily take the lead in morbid excitement, and impart to the disease an acute violence and character; but, in maladies of a more chronic kind, the seat of the affection may be in a texture that may not immediately disturb any of the vital organs: such an effect, however, might sooner or later ensue. In these instances, the excitability will be more particularly called into action in the affected part, evincing an accumulated degree of fibrous excitement and vascular distention independently of any generally increased action of the heart and arteries. Cases of disease from partial determination of excitement and vascular distention are amongst the most frequent, the least tractable, and most afflicting, of human maladies. They are obscure in their origin, slow in their progress, and they often insidiously disorganize and render incurable the affected part. Glandular diseases, those of vitiated secretions, dyspeptic derangement, and visceral congestions, are of this unperceived and untoward description.

The affections resulting from an unequal distribution of excitement and of the circulating fluids, however various in form and degree, can only be remedied by restoring the lost equality in those conditions of health, which ought naturally to exist. It is natural that the most healthful state of excitement and circulation should obtain; habit, as well as all suitable provisions for these effects, will powerfully conduce to secure the benefit of inherent regularity. When, therefore, disorder has arisen, the means of art for controling it will be aided by the strongest disposition which appropriate organization and established influence can afford. The general indication must be to equalize in the relative proportion that is natural the fibrous excitement and fluid circulation that are indispensable to health. Affections arising from either or both of these causes of disease are denoted as well by the situation of the disordered part as by the violence of the attending symptoms. If there be reason to suspect either venous plethora from visceral or other obstruction, or vascular fulness and consequent inflammatory action from accumulated blood and inordinate excitement, the measure of relieving the sanguiferous system by bleeding to a sufficient extent to disembarrass the heart and arteries of any impediment in duly distributing the circulating fluids, is obviously proper. This mode of remedy should be persisted in until the desired effect be produced; not by removing large quantities of blood at once, but by such a series of bleeding as the state of the arterial action would require, and such as would ultimately afford sufficient room in the sanguiferous system for an uninterrupted transmission of the circulating fluids. Density in the texture or consistency of the blood, with a coagulated serous surface, will evince strong arterial action, and that the prevailing excitement is so immoderate as to occasion an hurtful waste of vital power, and, by indefinite protraction, even to endanger irreparable and destructive disorganization in the situation where the diseased action may be going on.

The influence of cathartic medicine, by causing an additional afflux of fluids to the intestines, will greatly aid in diverting from the part that may be encumbered and disordered by an undue deposit of excitement and circulating fluids. The increased flow of fluids, as well as the augmented discharges that are obtained from the enteric and biliary secretions by the action of cathartic medicines, cannot fail to diminish general excitement, and to assist in obtaining the necessary depletion in the vascular system for re-instating a healthful transmission of the circulating fluids. Cathartic stimulus contributes also by its exciting influence over the whole system to equalize the distribution of excitement, and thus to relieve other, and even distant, parts from the pains and difficulties of a partial and undue exertion of vital power.

Sudorific or perspirative agency, by determining to the skin, would importantly further the aim of equalizing the distribution of excitement and of the circulating fluids. If the cutaneous exhalants, as well as every other portion of the minute division of the arterial system, were to be duly filled with the circulating fluids, the derivation thus effected from a part morbidly encumbered with them would necessarily afford great relief. In a natural and equal extension of the circulating function to the skin, the temperature of the body likewise comes to be so equalized as to be likely to acquire the healthy standard. This is a most important circumstance in all cases of disease, and particularly in instances of immoderate excitement and oppression from an unequal distribution of the circulating fluids. The cutaneous surface, with the bronchial structure of the lungs, may be considered as nature's grand outlet from the system of superfluous heat and vaporous or gazeous fluids. These excretions could not be retained without inducing morbid plenitude and temperature of the most menacing description. An open state of the skin, therefore, may be considered as an essential condition, if not a *sine qua non*, of health; and it must of course be of the utmost benefit in relieving and curing diseases. With a view to this advantage, the warm bath may be used with the best effects: its influence in rendering the skin freely pervious is very great, by subjecting the cutaneous exhalants to the two-fold agency of augmented heat, from the accumulated force of that which is incessantly escaping by insensible perspiration, and the additional warmth that is supplied by the high temperature of the bath. A gratefully tepid bath is better adapted for inducing beneficial perspiration than when used at a high temperature; as, in the one case warmth and vapour are passing off from the skin, whilst in the other they are detained by the excessive and stimulating influence of the accompanying heat. A fluid above the temperature of the skin imparts heat to its surface, but cannot convey it from thence; so that, pending the agency of the accumulated warmth of the bath, conjoined to that of the system, which is impeded from escaping, the heart and arteries are excited to increased action, and, were it to be protracted, injury might be done to either the morbidly irritable or positively diseased parts of the body.

Aqueous dilution, to the extent indicated by thirst, and in such proportion as may be necessary to supply the requisite fluidity for the circulating and secreting offices of life, and more particularly to attenuate and prevent hurtful concentrations of the various materials of which the circulating fluids are composed, is indispensably required. Dilution serves to remove what may be impregnated with effete or noxious substances, and also as a vehicle for the diffusion of temperature, and to promote the evaporation of redundant moisture and heat incessantly proceeding from the cutaneous surface. Watery liquids alone are real diluents; they furnish a mechanical medium through which the various substances necessary to animal life are distributed and transmitted over the whole frame. Small potations of slender liquids are well adapted in all cases of morbid excitement to wash off from the circulating fluids materials which if retained might be noxious, and to edulcorate as it were the remaining mass.

Blistering tends to equalize excitement and to invite an additional flow of fluids to the vesicated part; it may, therefore, be employed as a potent auxiliary in endeavouring to obtain the natural equilibrium of fibrous excitement and fluid circulation over the system, on which the healthy state essentially depends. It is known that morbid excitement is transferable,—that it may be either diminished or diverted by superinducing an additional exertion of vital power in the vicinage of the diseased action. Sometimes the institution of inflammatory excitement in a distant situation from the affected part will avail in mitigating and even subduing the affection. Vesication, setons, issues, caustics, and rubefacients, act on the same principle, and may be respectively used for the relief of parts suffering under accumulated excitement and a redundancy of the circulating fluids.

Brisk friction may be employed in a similar intention to that of other external stimulants. It excites the vessels immediately rubbed, and induces on them increased action, which may effect a salutary derivation from internal parts encumbered with undue irritation and fluidity. The medical or sanitive power of friction is very great; it energizes and brings into vigorous action the parts directly subjected to its operation; and often, by sympathy or consent, as in the instances of the stomach and intestines, renovates and re-establishes the healthy power of those organs. It is a remedy of great importance in all chronic affections of the abdominal viscera, and its use should be only limited in frequency and continuance by the circumstance of the skin being rendered too sore by its employ to admit of its being farther performed.

The diseases resulting from an undue distribution of excitement and of the circulating fluids cannot be overcome by stimulating agency. To awaken additional exertion of vital power under such circumstances would be to exasperate the prevailing disease. The curative object is to make sufficient room in the sanguiferous vessels, by suitable evacuations, to admit of the circulating fluids being freely distributed in relative proportion over the whole system. When this has been effected, visceral oppression, whether of the brain, lungs, liver, or any other organ, will cease, and the various morbid sympathies associated with such diseased derangement will likewise terminate.

The benefit accruing from the removal of the cause of disease, is alone strictly and permanently curative. It is not the alleviation of distempered sensibility,—it is not the torpid respite of previous exhaustion,—it is not the transient and deceptious amendment, of momentary influence; but it is the radical correction of the diseased state that effects a lasting cure. Generally speaking, the diseased state produces sufficient excitement to ascertain what may be accomplished by stimulating agency. The natural effort made by the energies of the affected part is often availing; it frequently overcomes the cause of disease: but when it has failed, and accumulated mischief and difficulty are taking place, the resources of the medical art should be sedulously employed to restrain and cure the increasing malady.

Dietetic regulations, with a view to secure at once unimpeded digestion, adequate nourishment, an active state of bowels, particularly of the duodenal portion of the intestinal canal, with which the biliary secretion is intimately associated,—occasional vascular depletion, when indicated by arterial fulness and other characteristic symptoms of either general or partial plethora,—congenial temperature,—atmospheric purity,—and suitable bodily exercise, are amongst the most powerful means of effecting a complete and durable recovery from the various morbid derangements arising from undue fibrous excitement, and from the circulating fluids being unequally distributed over the system.

*Taunton; July 30th*, 1822.

